# Operative Measures in Glaucoma

**Published:** 1881-12

**Authors:** Charles W. Hickman

**Affiliations:** Augusta, Ga.; Lecturer on Eye, Ear and Throat Diseases in the Medical Department of the University of Georgia, Augusta


### OPERATIVE MEASURES IN GLAUCOMA.

BY CHARLES W. HICKMAN, M.D., AUGUSTA, GA.
Lecturer on Eye, Ear and Throat Diseases in the Medical Department of the University of Georgia, Augusta.

One of the most distressing affections with which the surgeon has to deal is that which we commonly understand as "glaucoma." While in the main, according to statements, this disease is not so often encountered in our own country as in Europe, yet we meet with it frequently enough to keep us constantly on our guard. Those of any experience in ocular disorders are aware of the serious nature of the affection, as well as the danger attending any delay of active measures for its treatment; indeed there are times when promptness alone will save our patient from certain blindness.

Although many peculiarities of this disease, such as the stony hardness of the globe, dilated pupil, hazed cornea, and agonizing attacks of neuralgia, and above all, the greenish reflex from the interior of the eye, have been long recognized, as well as the unhappy termination of this so-called amaurosis, yet it is only of recent date that we have been in a position to arrest the wasting vision in its course, or to afford relief from the accompanying paroxysms of pain.

If we have to thank the genius of Helmholtz for the discovery of the ophthalmoscope, enabling us to gain more positive information in regard to the nature of this affection, to see the peculiar cup shape of the optic nerve, the enlarged and tortuous veins, the pulsation of the vessels and their dipping over the excavation, being, as it were, lost to view; how much more do we owe our still greater benefactor, von Graefe, for the only reliable means of successfully dealing with this baneful disease. The eyes that have been saved, and the ceaseless hours of pain that have been spared since von Graefe first instituted operative measures for the relief of glaucoma can scarcely be estimated.

Although so much has been said for and against such measures, some surgeons loudly extolling their virtues, and others upholding them in a very indifferent manner, if at all, yet if we but bear in mind the few broad principles which experience has shown to be trustworthy, we cannot go far astray. At any rate, even though our efforts are not crowned with success, the comforting assurance remains that everything within the present knowledge of surgical science has been done for the relief of our patient.

Let us remember, first, that if the disease is left to itself, blindness is the inevitable result; secondly, that with the exception of the little good to be obtained from esserine, drugs of any kind are utterly valueless; and, thirdly, that of all operative procedures none are so sure or so trustworthy as iridectomy. Von Graefe faithfully tried, time and again, tapping of the anterior chamber, and there is scarcely a surgeon of any repute in ocular disorders who has not as faithfully tested its powers. Almost the universal opinion in reference to it has been, that a few cases are occasionally relieved, but in the majority the relief, if given at all, is only temporary. The disease is apt to return with increased vigor, and each return is attended with a perceptible diminution of vision. The relief from iridectomy, however, is permanent.

Next, we should remember that when the disease is well under way, the sight which is lost cannot be restored. It may be arrested in its course; what is left of the power of vision may be saved, and at times some little improvement even be expected. In addition to this result, in the arrest of the disease in its progress towards blindness, comes also an entire cessation of the attacks of neuralgia.

Where cases are presented to us in which blindness has already set in, we cannot hope to restore the sight, but still we should not hesitate to operate, if only for the relief of the pain. Patients frequently tell us in such instances that they are greatly happy, although their sight has not been restored, to be relieved of the intense pain they suffer.

Then again, we should remember that the iridectomy should differ somewhat from that for other purposes, in being made large; the incision with a broad iridectomy knife passing through the sclerotic into the anterior chamber, the iris forceps gently carried through the wound, and a large piece of iris withdrawn and snipped off quite close up to its insertion near the sclerotic. Should we prefer, instead of using the iridectomy knife, we may skirt the upper margin of the anterior chamber with a delicate, narrow, von Graefe's cataract knife, giving us the advantage of a larger opening, but the disadvantage of not so easily controlling the flow of aqueous.

With these rules ever before us, notwithstanding the fact that we cannot always look for success, still in a very large proportion of cases, not only may much good be obtained from operative measures in this disease, but our results at times are not far short of brilliant. As a matter of course, a candid statement of the condition of the case and probable results, should at all times be made to the patient.

Without going into an extended list of cases, a few may be mentioned in illustration. If they will in any way aid in extending confidence in the preceding principles, and thereby affording relief to many sufferers, the author will indeed feel grateful.

The wife of a laborer, A. H., came under notice for violent pain in both eyes, which had for a considerable period rendered her life unendurable. During this time her sight was gradually but surely fading away. The pupils were dilated to some extent, and attended with a greenish reflex from the interior of the eyes. Ophthalmoscopic examination revealed cupping of both optic nerves, and of the bluish shades so common to glaucoma, the retinal vessels terminating at the edge and lost to view over the surface. The importance of an early operation being strongly urged, and to which consent was readily given, an upward iridectomy was made in both eyes, resulting in entire cessation of pain, the arrest of wasting sight, and attended even with some little improvement in the power of vision.

Sarah S., a servant aged 55, was seen for paroxysmal pains in her left eye, severe in character, causing her much suffering, and which had reduced her vision to almost nothing. Although the fundus could not be clearly seen, yet the stony hardness of the ball, and other symptoms, pointed clearly to glaucoma. An iridectomy having been advised and made (upward), complete cessation of pain resulted, although, as is to be expected in such cases, the improvement in sight was only slight.

I mention the next case as showing that operative measures do not always yield such gratifying results.

Miss Annie C., a lady of delicate constitution and nervous temperament, sought relief for pains in both eyes, which had been at times quite severe, and attended by a gradual diminution of vision. The tension of the eyeballs was considerable in degree, pupils dilated and the greenish reflex quite marked, though no appreciable amount of cupping was to be seen at the time. A candid statement of what she had to expect having beem made to her, and the trial of an early operation urgently insisted upon, an upward iridectomy was performed in both eyes, but not attended by any very marked relief, such as every operator anxiously hopes for even in these cases of chronic glaucoma.

The next case is that of Mr. H., a farmer, who presented himself for severe neuralgia of his right eye, paroxysms of pain coming on, so intense in character as to render his enduring them almost impossible, except under the influence of opiates. The eyeball was bard and pupil dilated, yet no cupping, but with vision considerably reduced. As he desired to return home as quickly as possible, influenced by statements received of beneficial effects obtained from tapping the anterior chamber, notwithstanding so much had been said against it, I was induced to make the attempt. As no harm could be done, any way, I was anxious to discover whether this procedure would, in this case, result in good. The nature of this operation, so much less in degree, having been explained to him, as well as the probability of his having soon to return for the performance of an iridectomy for permanent relief, the needle was introduced into the anterior chamber to allow the aqueous to flow off, a drop or two of atropine put into the eye (2 grs. to ℥1), a bandage applied, and the patient soon allowed to return home. Up to the present time, ten months having elapsed, the accounts received from him are that the pain has entirely disappeared, and further, to use his own words, "he sees better out of that eye than his other."

